# Imbalanced Expression of Tau and Tubulin Induces Neuronal Dysfunction in *C. elegans* Models of Tauopathy

**DOI:** 10.3389/fnins.2018.00415

**Published:** 2018-06-20

**Authors:** Tomohiro Miyasaka, Yuki Shinzaki, Satomi Yoshimura, Sawako Yoshina, Eriko Kage-Nakadai, Shohei Mitani, Yasuo Ihara

**Affiliations:** ^1^Department of Neuropathology, Faculty of Life and Medical Sciences, Doshisha University, Kyoto, Japan; ^2^Department of Physiology, School of Medicine, Tokyo Women’s Medical University, Tokyo, Japan; ^3^Graduate School of Human Life Science, Osaka City University, Osaka, Japan

**Keywords:** tau, tauopathy, microtubule, tubulin, neurodegeneration, *C. elegans*, Alzheimer’s disease

## Abstract

Tauopathy is a type of dementia defined by the accumulation of filamentous tau inclusions in neural cells. Most types of dementia in the elderly, including Alzheimer’s disease, are tauopathies. Although it is believed that tau protein abnormalities and/or the loss of its functions results in neurodegeneration and dementia, the mechanism of tauopathy remains obscure. Loss of microtubules and/or tubulin is a known consequence of tau accumulating in neurons in Alzheimer’s disease. In other words, there is an excess level of tau relative to tubulin in tauopathy neurons. To test whether this imbalance of tau and tubulin expression results in the neurotoxicity of tau, we developed several transgenic *C. elegans* lines that express human tau at various levels in pan-neurons. These worms showed behavioral abnormalities in a tau expression-dependent manner. The knockdown of a tubulin-specific chaperon, or a subset of tubulin, led to enhanced tau toxicity even in low-expressing tau-transgenic worms that showed no abnormal behaviors. In addition, the suppression of tau expression in tubulin knockdown worms rescued neuronal dysfunction. Thus, not only the overexpression of tau but also a reduction in tubulin can trigger the neurotoxicity of tau. Tau expressed in worms was also highly phosphorylated and largely bound to tubulin dimers rather than microtubules. Relative amount of tubulin-unbound tau was increased in high-expressing tau-transgenic worms showing tau toxicity. We further demonstrated that tau aggregation was inhibited by co-incubation of purified tubulin *in vitro*, meaning sufficient amounts of tubulin can protect against the formation of tau inclusions. These results suggest that the expression ratio of tau to tubulin may be a determinant of the tauopathy cascade.

## Introduction

In certain types of neurodegenerative diseases, affected neurons or glial cells have filamentous tau inclusions called neurofibrillary tangles, neuropil threads, and glial fibrillary tangles (Serrano-Pozo et al., 2011; Spillantini and Goedert, 2013). These disorders, which constitute the majority of age-dependent dementia, are called tauopathies and include Alzheimer’s disease (AD), frontotemporal dementia, progressive supranuclear palsy, and corticobasal degeneration. The severity of dementia in tauopathies has been shown to correlate with the abundance of tau inclusions and the extent of neuronal loss (Gomez-Isla et al., 1997; Delacourte et al., 1999). Furthermore, the pathogenic mutations in the tau gene have been identified from genetic studies of familial tauopathy, FTDP-17 (Iqbal et al., 2016). These findings indicate that changes in tau and its function lead to neurodegeneration and dementia.

Numerous studies revealed that pathological tau inclusions are composed of insoluble filaments, paired helical filaments (PHF) and straight filaments (Grundke-Iqbal et al., 1986; Greenberg and Davies, 1990; Lee et al., 1991; Fitzpatrick et al., 2017). This pathologically deposited tau is abnormally phosphorylated to a greater extent than physiological tau (Khatoon et al., 1992; Hanger et al., 2009). Since this abnormal phosphorylation disrupts the ability of tau to promote microtubule (MT) assembly (Yoshida and Ihara, 1993), it has been considered a key element of the pathology that underlies tauopathies. However, the pathological significance of the phosphorylation on individual sites of tau remains unclear. Other abnormal modifications, such as ubiquitination, acetylation, glycation, isomerization, and truncation, do not occur as often in physiological tau but were also identified (Miyasaka et al., 2005b; Iqbal et al., 2016). In contrast to the huge filamentous inclusions, it is currently assumed that the small soluble aggregates called oligomers are an essential part of the tau toxicity or substance that propagates tau pathology (Goedert et al., 2017; Shafiei et al., 2017). However, it is also suggested that unaggregated tau can affect neuronal function (Miyasaka et al., 2016; Xie and Miyasaka, 2016). Thus, the definitive trigger of tau, as well as which type of tau is toxic, is currently unknown.

Loss of MTs and/or tubulin is an invariable feature of tau-accumulating neurons in AD (Terry et al., 1964; Cash et al., 2003; Zhang et al., 2015). This inverse relationship is reproduced in the affected neurons of tauopathy animal models (Tatebayashi et al., 2002; Miyasaka et al., 2005a, 2016). Because the physiological function of tau is known to promote the assembly of tubulin or to stabilize MTs by binding to them, it is believed that the hyper-phosphorylation of tau may lead to MT loss (Bodea et al., 2016; Wang and Mandelkow, 2016). However, the finding that tau-knockout mice did not show obvious developmental abnormalities or brain function abnormalities, suggests that MT loss cannot simply be explained by a lack of tau (Harada et al., 1994; Kimura et al., 2014). Recently, we showed that the hyper-phosphorylation of tau in hypothermic brains did not directly detach tau from MTs and that forced MT destruction induced simultaneous tau liberation and phosphorylation in cultured cells (Planel et al., 2008; Miyasaka et al., 2010). Furthermore, a reduction of MTs was found in the neurons of AD brains regardless of neurofibrillary tangle formation (Cash et al., 2003). These findings suggest that MT loss (or tubulin reduction) is not a passive event and is involved in the early pathogenesis of tauopathy.

We speculated that the amount of the tubulin in neural cells determines tau toxicity and that excess tau relative to tubulin triggers the tauopathy cascade. To confirm that the imbalanced expression of tau and tubulin can induce tauopathy, we developed several lines of transgenic *C. elegans* that express various levels of human tau pan-neuronally, and then, we assessed the effects of tubulin knockdown.

## Materials and Methods

### Development and Maintenance of Worm Strain

Development and maintenance of integrant worm lines were performed as previously described (Xie et al., 2014; Miyasaka et al., 2016). Briefly, human tau or DsRed cDNA was subcloned into the site downstream of “pan-neuronal” *unc-119* promoter (punc-119) of pFXneo-punc119 vector (Maduro and Pilgrim, 1995). The transgenes were injected into N2 together with a marker, pFXneo-Pges-1::EGFP. Germline transformation and generation of extrachromosomal arrays in C. elegans strains were performed according to the standard protocol (Mitani, 1995). Stable tau-transgenic (Tg)-lines, generated by UV irradiation, were backcrossed to N2 five times before analysis. Tg-strains used here were as follows: Mock-Tg (tmIs388), WT4R(L)-Tg (tmIs389), WT4R(H)-Tg (tmIs390), WT4R(ExH1)-Tg (tmIs763), and WT4R(ExH2)-Tg (tmIs765). For RNAi, tau-Tg worms were crossbred with *rrf-3* mutant lines (Mock-Tg/rrf-3 and WT4R(L)/rrf-3). For *mec-7* or *mec-12* knockout, mec-7 (e1506) or mec-12 (tm5083) mutant lines were used. All *C. elegans* strains were maintained on a nematode growth medium (NGM) plate spread with E. coli OP50 under standard conditions (Miyasaka et al., 2016).

### Feeding RNAi

The RNA interference of C. elegans by feeding with dsRNA-expressing *E. coli.* was performed as previously described, with minor modifications (Kage-Nakadai et al., 2016). Complementary DNA fragments encoding human tau (748–1326 bp; corresponding to 2N4R isoform tau cDNA), *K07H8.1* (*tbce-1*; 1066–1298 bp), and *mec-12* (619–1058 bp) were subcloned into the multiple cloning site of the L4440 vector and transformed to the HT115 (DE3) *E. coli.* strain. The desired double-stranded RNA (dsRNA) was produced by bi-directional expression induced by 1 mM IPTG. The bacteria expressing dsRNA were spread on NGM media supplemented with ampicillin and IPTG. Synchronously cultured worms were grown on these RNAi plates for 4 days and subjected to behavioral and biochemical analyses. To ensure efficient knockdown in neuronal cells, the worm lines crossbred with *rrf-3* were used for all RNAi experiments (Simmer et al., 2002).

### Behavioral Analyses

A touch assay was performed as previously described (Miyasaka et al., 2005a). Briefly, on the forth day after hatching the worms were isolated onto new 3.5-cm plates, and their escape reactions were assessed in response to gentle touch with an eyelash under a stereoscopic microscope. The number of responses to 10 touch trials, five for the anterior plus five for the posterior, were counted. Uncoordinated movements (Unc) were also assessed under a microscope. The severity of Unc was scored as follows: normal, wide bending with fast movement (2 point); Unc, slow movement (1 point); severe Unc, negligible movement (0 point). Twenty worms were used in each assay for each experiment, and the experiment was performed three to four times. Thus, 60–80 worms were assessed for each line. For the abnormality in *tbce-1* knockdown, the severities of Unc of all worms grown on knockdown plates were scored as follows: normal, wide bending with fast movement (2 point); Unc, slow movement (1 point); severe Unc, negligible movement and burst (0 point).

### Biochemical Analyses

For biochemical analysis, worms were grown on NGM plates at large rectangular plates (No. 2 square schale, Eiken chemical co ltd., Tokyo, Japan). Synchronized worms were harvested in M9 buffer and pelleted by brief centrifugation. After washing twice with M9 buffer (22 mM KH_2_PO_4_, 42 mM Na_2_HPO_4_, 85 mM NaCl, and 1 mM MgSO_4_), the worm pellets were weighed and stored at -80°C. Total worm lysates were prepared as previously described (Miyasaka et al., 2016). The worm pellets were sonicated in sodium dodecyl sulfate (SDS) sample buffer (80-mM Tris-HCl, 2% SDS, 10% glycerol, 1% 2-mercaptoethanol, pH 6.8) and cleared by ultracentrifugation at 150,000 × *g* for 10 min at 20°C. The protein concentration for each sample was verified by Coomassie Brilliant Blue (Wako Pure Chemical Industries, Ltd., Osaka, Japan) staining on SDS-poly acrylamide gel electrophoresis (PAGE). Soluble fractions were subjected to SDS-PAGE followed by Western blotting. The MT-binding of expressed tau was analyzed as previously described (Miyasaka et al., 2016). Freshly harvested worms were homogenized in MS buffer (0.1 M MES, 1 mM EGTA, 1 mM MgSO_4_, 2 mM DTT, and 0.5% Triton X-100, pH 6.8) containing 20 μM Taxol, 2 mM guanosine triphosphate (GTP), and protease inhibitors (cOmplete^®^, Sigma-Aldrich, St. Louis, MO, United States) and phosphatase inhibitors (1 mM Na3VO4, 1 mM NaF, 1 mM okadaic acid, and 1 mM b-glycerophosphate) at room temperature and then immediately chilled. After brief centrifugation, soluble fractions were centrifuged at 100,000 × *g* for 15 min at 2°C to obtain the soluble free tubulin and insoluble MT fractions. MT-unbound tau in free tubulin fraction was immunoprecipitated with H-150 anti-tau antibody bound on ProteinG-conjugated Dynabeads (Thermo Fisher Scientific Inc., Waltham, MA, United States) at 4°C for 3 h. After washing with MS buffer, bound proteins on the beads were solubilized in SDS-sample buffer. The amount of tau and tubulin were analyzed by Western blotting.

### Purification and Dephosphorylation of Tau Expressed in Worms and MT-Binding Assay

Purification and dephosphorylation of tau and an MT-binding assay were performed as previously described with minor modifications (Xie et al., 2014). Four-day-old WT4R-Tg worm pellets were homogenized in ×10 volume of Tris-saline buffer (TS; 50 mM Tris, 150 mM NaCl, pH 7.6) containing protease and phosphoatase inhibitors described above. After ultra-centrifugation at 120,000 × *g* for 15 min at 2°C, the soluble fractions were adjusted in 0.5 M NaCl and 2% 2-mercaptoethanol and heated at 100°C for 5 min. After the precipitates were removed by brief centrifugation, the soluble (heat-stable) fractions were precipitated by 50% ammonium sulfate. After centrifugation at 20,000 × *g* for 15 min at 2°C, the resultant pellets were re-solubilized and incubated with or without Lambda protein phosphatase (New England BioLabs, Inc., Ipswich, MA, United States) for 30 min at 30°C. Dephosphorylated and non-dephosphorylated tau were purified by using both heat and ammonium sulfate precipitation as described above. For the preparation of worm MT, fresh Mock-Tg worms were homogenized in ×5 volume of MS buffer containing protease and phosphatase inhibitors and centrifuged at 120,000 × *g* for 15 min at 2°C. Then, the soluble fraction was incubated with 20 μM Taxol and 2 mM GTP for 30 min at 25°C. After the addition of 0.5 M NaCl to remove the endogenous MAPs, polymerized MTs were collected by ultra-centrifugation and re-suspended into MS buffer containing Taxol and GTP. After the re-binding of purified tau on MTs at 25°C for 20 min, MT-unbound and MT-bound fractions were separated by ultra-centrifugation.

### Morphological Analyses

Immunocytochemical analyses were performed by using the freeze-cracking method (Miyasaka et al., 2005a). Cracked worms put on PDL (poly-d-lysine)-coated glass slides were immersed in pre-cooled MeOH followed by hydration and permeabilization with 0.1% Triton X-100 in TS. After the worms were soaked with 10% goat serum in TS, the specimens were incubated with 1% BSA in TS containing primary antibodies. Bound antibodies were visualized using Alexa-conjugated secondary antibodies (Molecular Probes, Inc., Eugene, OR), and observed under an LSM700 microscope (Carl Zeiss Inc., Jena, Germany).

### Drug Treatment

Trimethylamine-N-oxide (TMAO) was dissolved in H_2_O and stored in -20°C. For treatment, the compound was diluted with M9 buffer and directly added onto NGM plate. Worms were synchronized and grown on drug-containing plates for 3 days and subjected to biochemical analyses.

### *In Vitro* Tau Aggregation Assay

Purification of recombinant tau corresponding to the 0N4R isoform and *in vitro* tau aggregation assays, were performed as previously described (Xie et al., 2014, 2015). Briefly, the recombinant tau 0N4R isoform expressed in *E. coli* [BL21(DE3)] was solubilized in homogenization buffer (50 mM PIPES, 1 mM EGTA, 1 mM DTT, and pH 6.8) with protease inhibitors and charged onto a phosphocellulose column (P11, Whatman). Tau protein fraction eluted in 0.1–0.3 M NaCl was precipitated by ammonium sulfate precipitation. The resultant pellet was re-solubilized by homogenization buffer containing 0.5 M NaCl and 1% 2-mercaptoethanol and fractionated by heat stability. The heat-stable fraction was further purified using reverse-phase HPLC (Cosmosyl protein-R, Nacalai tesque, Kyoto, Japan). Purified porcine tubulin was purchased from Cytoskeleton Inc. (Denver, CO, United States). Purity and protein amount were analyzed by SDS-PAGE followed by Coomassie brilliant blue staining. For *in vitro* aggregation, 60 mg/ml of heparin was mixed with 10 mM purified tau, 100 mM NaCl, 10 mM thioflavine-T (Th-T), 10 mM HEPES (pH 7.4), and with/without tubulin. The time-dependent changes in Th-T fluorescence were measured at 465–635 nm (excitation) and 535–625 nm (emission) for 3 days. After incubation, the mixtures were centrifuged at 100,000 × *g* for 15 min before or after 1% Sarkosyl, treatment. The fractions were analyzed by SDS-PAGE followed by CBB staining and Western blotting.

### Antibodies and Compounds

The antibodies used here were as follows: pool-2 (anti-pan-tau, a generous gift of Dr. H. Mori), HT7 (anti-pan-tau, Innogenetics), H-150 (anti-human-tau, Santa Cruz Biotechnology, Dallas, TX, United States), AT8 (anti-phospho-tau, Thermo Fisher Scientific Inc.), tau-1 (anti-dephospho-tau, Merck Millipore, Billerica, MA, United States), PHF-1 (anti-phospho-tau, a generous gift of Dr. P Davies), pS262 (anti-phospho-tau, Sigma-Aldrich, St. Louis, MO, United States), DM1A (anti-α-tubulin, Sigma-Aldrich), KMX-1 (anti-β-tubulin, Merck Millipore), anti-mec-12 (6-11B1, Sigma-Aldrich), 1A4 (anti-α-actin, Sigma), and 6C5 (anti-GAPDH, Abcam). Taxol and trimethylamine-N-oxide were purchased from Sigma-Aldrich. Other materials were purchased from Nachalai tesque unless otherwise specified (Kyoto, Japan).

### Statistical Analysis

All statistical analyses were performed using IBM SPSS statistics 25 (IBM, Armonk, NY, United States). Analysis of variance (ANOVA) was used for comparisons made between three or more groups, followed by Tukey’s *post hoc* test. All data were presented as the means ± SEM. In all cases, *P* values lower than 0.05 were considered as significant.

## Results

**FIGURE 1:**
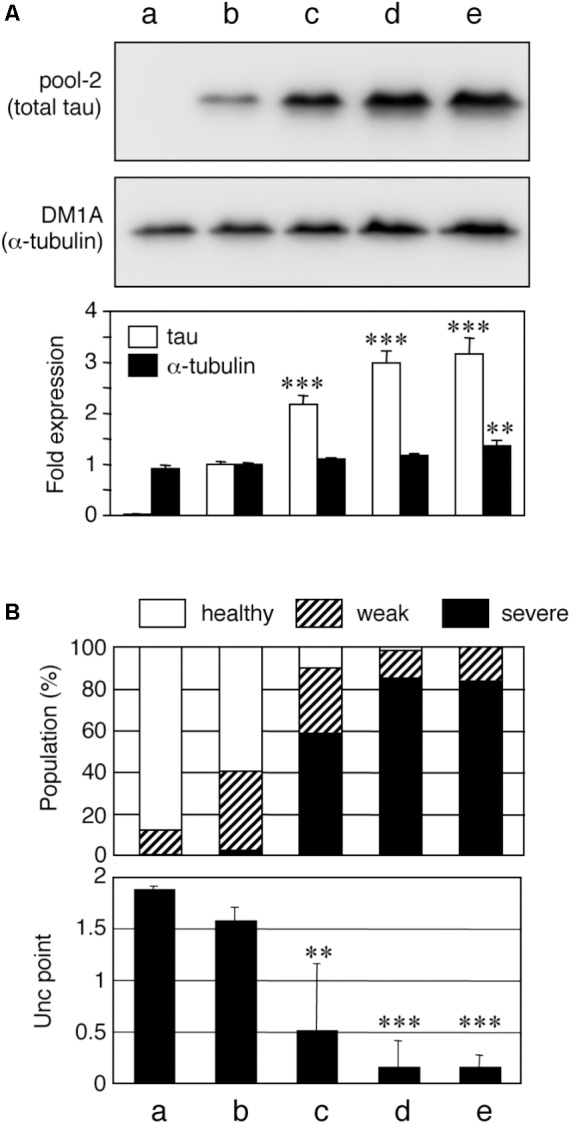
Tau level-dependent neuronal dysfunction in WT4R-tau-Tg worms. Mock-Tg **(a)**, WT4R(L)-Tg **(b)**, WT4R(H)-Tg **(c)**, WT4R(ExH1)-Tg **(d)**, and WT4R(ExH2)-Tg **(e)** worms were grown on NGM plates and subjected to **(A)** Western blotting of tau (pool-2) and tubulin (DM1A) in the total lysate. Expression levels were quantified and shown against WT4R(L)-Tg (Lower panel). Average of four independent experiments are shown (means ± SEM, *n* = 4). Statistical significance was analyzed by Tukey’s *post hoc* test (^∗∗^*p < 0.01*, *^∗∗∗^p < 0.001*, *vs. WT4R(L)-Tg*). **(B)** The behavioral analysis of uncoordinated movement in each worm line was analyzed. Upper panel shows the population of healthy (open), weak Unc (hatched), and severe Unc (closed) worms (*n* = 60) in each line. Lower panel shows that the severity of Unc as described in Materials and Methods section. Average scores of three independent experiments are shown (means ± SEM, *n* = 3). Statistical significance was analyzed by Tukey’s *post hoc* test (^∗^*p < 0.05*, ^∗∗^*p < 0.01*, *^∗∗∗^p < 0.001*, *vs. Mock-Tg*).

To date, a number of Tg mouse lines have been developed (Noble et al., 2010), with varying neurological phenotypes. Although some of the unique phenotypes may be caused by the promoter used for exogenous tau expression in particular lines, it is generally understood that the expression level of tau correlates with the severity of the pathology in Tg mice. To probe whether the amount of tau expression affects its neuronal phenotype, we developed several lines of tau-expressing worms using the pan-neuronal punc-119 promoter (Xie et al., 2014; Miyasaka et al., 2016). Integration of extrachromosomal arrays in the worm genome allows for the introduction of various numbers of transgene copies (Mitani, 1995). We isolated four independent integrant human wild-type 0N4R-tau-Tg lines with different expression levels (**Figure 1A**). WT4R(H)-Tg, WT4R(ExH1)-Tg, and WT4R(ExH2)-Tg lines express tau at 2.17 ± 0.17, 2.98 ± 0.24, and 3.16 ± 0.31 fold than WT4R(L)-Tg respectively. We also found the amount of α-tubulin expression was slightly but significantly higher in WT4R(Ex2)-Tg line. As shown in **Figure 1**, WT4R(L)-Tg worms showed minimal Unc but the WT4R(H)-Tg worms expressing three times more tau had a more severe Unc phenotype as previously described (Miyasaka et al., 2016). Furthermore, most of the lines with extremely high tau expression levels, WT4R(ExH1)-Tg and WT4R(ExH2)-Tg, suffered from severe Unc and were immobile (**Figure 1B**). Therefore, in these lines, the severity of Unc seemed to be correlated with the expression levels of exogenous tau.

**FIGURE 2:**
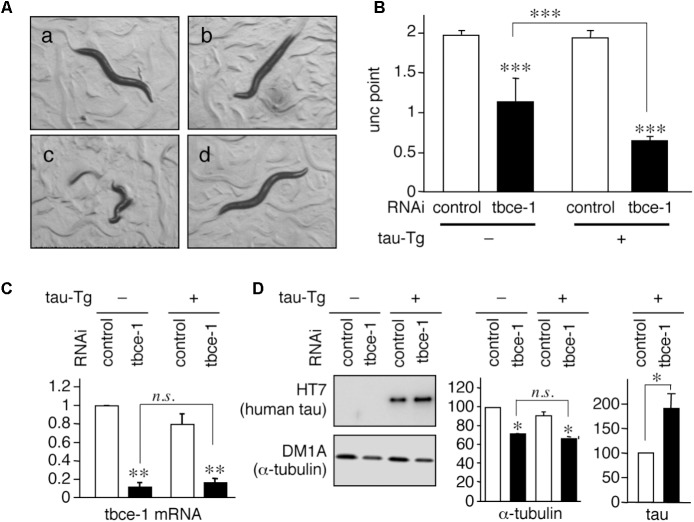
Tau expression enhances uncoordinated movement induced by *tbce-1* gene knock down. **(A)** WT4R(L)-Tg/rrf-3 **(a–c)** and Mock-Tg/rrf-3 worms **(d)** were grown on L4440::T (a; control) or L4440:: *tbce-1* (**b–d**; *tbce-1*) RNAi plates from embryo to 3 days after hatch. Rrf-3 background worms grown on *tbce-1* RNAi plates showed Unc **(b)** or a frequent burst phenotype **(c)**. **(B)** The severity of Unc was assessed as described in Materials and Methods section. Average scores of three independent experiments are shown (means ± SEM, *n* = 3). WT4R(L)-Tg/rrf-3 worms showed a significantly higher sensitivity to tubulin chaperon knockdown. **(C)** The levels of *tbce-1* mRNA were quantified by quantitative real time RT-PCR (means ± SEM, *n* = 3). *tbce-1* mRNA is suppressed to less than 25% compared to controls, regardless of tau expression. **(D)** The levels of tau and α-tubulin were analyzed by Western blotting. The percent expression of control worms is shown (means ± SEM, *n* = 3). Statistical significance was analyzed by Tukey’s *post hoc* test (^∗^*p < 0.05*, ^∗∗^*p < 0.01*, and *^∗∗∗^p < 0.001*) or Student’s *t*-test (^∗^*p < 0.05*).

When tau expression levels were greater than that of in WT4R(H)-Tg, neurotoxicity was present. Pathological studies indicated that the amount of MTs and/or tubulin is inversely correlated with tau-accumulating neurons (Terry et al., 1964; Cash et al., 2003; Zhang et al., 2015). Therefore, we hypothesize that the amount of tubulins may determine a threshold of neurotoxicity induced by tau. To test whether the expression level of tubulin affects tau toxicity, we sought to reduce tubulin expression in tau expressing worms. Because there are nine α-tubulin genes and six β-tubulin genes in C. elegans (Gogonea et al., 1999), a direct knockdown of a single tubulin isotype may not be sufficient to reduce the level of total tubulin in neurons. Therefore, we chose the *tbce-1* gene, a homologue of human tubulin-folding cofactor E (*tbce*), a tubulin chaperon needed for *de novo* synthesis of α-tubulin (Tian and Cowan, 2013; Nithianantham et al., 2015). A missense mutation of the Tbce gene is identified in the spontaneous mouse model of progressive motor neuronopathy (Bommel et al., 2002; Martin et al., 2002), indicating that the functional decline of TBCE can induce MT abnormality in neurons. In C. elegans model, target genes can be knocked down easily by feeding E. coli expressing sence/antisense RNA *in vivo*. As shown in **Figure 2**, *tbce-1* knockdown by feeding-RNAi resulted in the reduction of α-tubulin and induced abnormal Unc behavior. In severe cases, the worms showed complete immobility and burst (**Figure 2A**). The severity of unc was significantly increased in human-tau expression (**Figure 2B**). *tbce-1* knockdown showed reduced expression of α-tubulin (**Figures 2C,D**). Intriguingly, *tbce-1* knockdown increased tau expression (**Figure 2D**). Thus, the low expression of tau can affect neuronal functions when the expression of tubulin is reduced.

**FIGURE 3:**
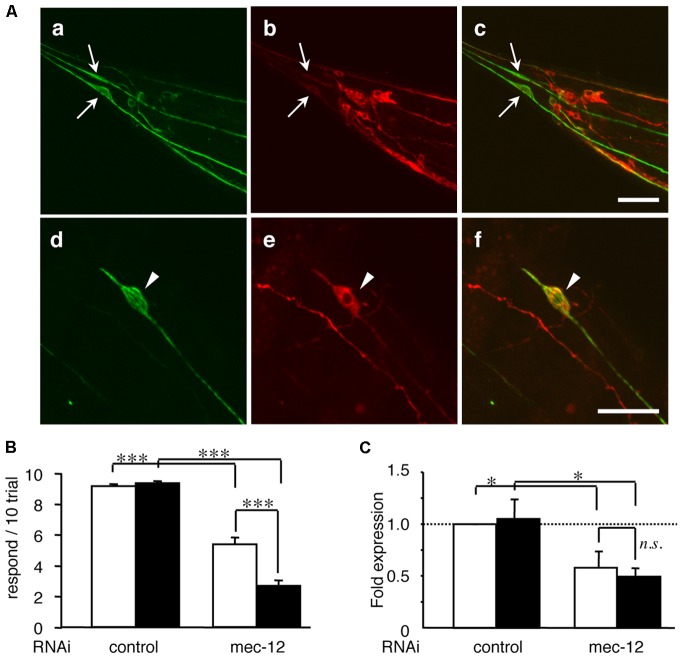
Tau expression in mechanosensory neurons exacerbates touch sense abnormality induced by tubulin knockdown. **(A)** WT4R(L)-Tg/rrf-3 **(a–c)** and Mock-Tg/rrf-3 **(d–f)** worms were subjected to immunolabeling using 6-11B1 (anti-mec-12; **a** and **d**), and pool-2 (anti-tau; **b** and **e**). Merged views are also shown **(c** and **f)**. Human tau was expressed in as pan-neuronal pattern (**b** and **e**) including PLMs (arrows in **a–c**) in lumber ganglia and ALMs (arrowheads in **d–f**) in the abdomen. Scale bars = 30 μm. **(B)** Mock-Tg/rrf-3 (open) and WT4R(L)-Tg/rrf-3 (solid) worms were grown on L4440::T (control) or L4440::mec-12 RNAi plates from embryo to 4 days after hatch. Touch sensitivity was analyzed as described in the Materials and Methods section (means ± SEM, *n* = 60). **(C)** The expression of *mec-12* mRNA in the indicated worms was quantified by quantitative real time RT-PCR (means ± SEM, *n* = 3). Statistical significance was analyzed by Tukey’s *post hoc* test (*^∗^p < 0.05*, *^∗∗∗^p < 0.001*).

To address whether tubulin knockdown directly enhances tau-induced neuronal dysfunction, we reduced the expression of *mec-12*, an isotype of α-tubulin specifically expressed in touch neurons (Fukushige et al., 1999), in WT4R(L)-Tg/rrf-3 worms, which express human-tau (**Figure 3A**). This is probably the only *in vivo* experimental model in which the relationship between specific tubulin isotype and neural function is strictly corresponded. Knocking-down *mec-12* in wild-type worms showed touch sense abnormality (**Figures 3B,C**), a phenotype of *mec-12* mutant worms. When the *mec-12* RNAi was applied to Mock-Tg and WT4R(L)-Tg worms in the *rrf-3* background, we found that the touch sense abnormality was significantly exacerbated in the WT4R(L)-Tg line (**Figures 3B,C**). Control RNAi (**Figures 3B,C**) on WT4R(L)-Tg/rrf-3 worms did not cause any tactile abnormalities. Thus, α-tubulin downregulation alone can cause abnormalities in touch neurons, which are enhanced by the expression of human-tau. We addressed whether these exacerbations were specific to tau and found that the toxicity of tubulin knockdowns or tubulin chaperon knockdowns was not enhanced by DsRed expression by the same *Unc-119* promoter (data not shown). Furthermore, the knockdown of genes unrelated to tubulin or MTs, including *gfp*, *far-3*, *mtl-1*, and *col-149*, did not show any abnormalities in tau-Tg worms (data not shown).

**FIGURE 4:**
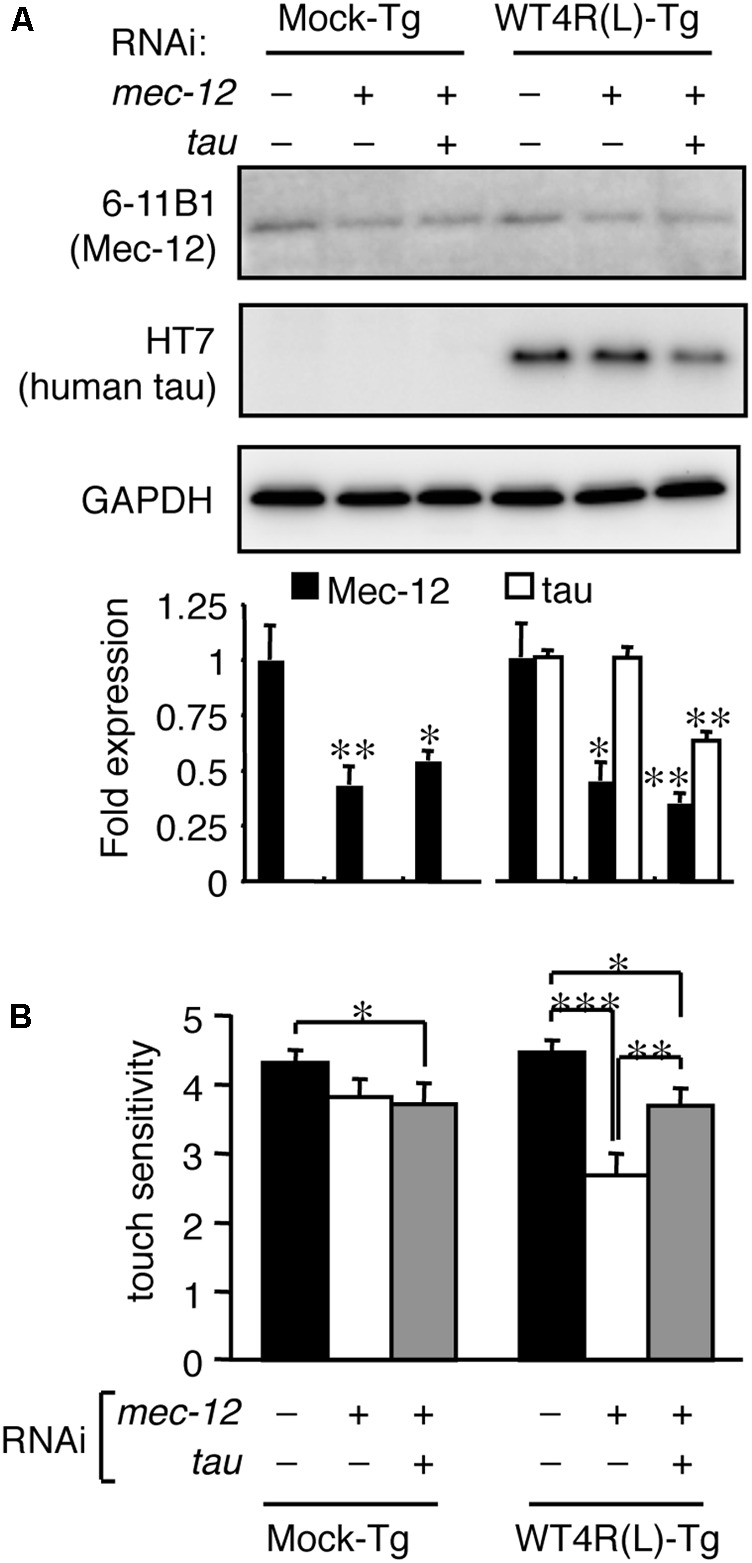
Tau reduction rescues the neuronal dysfunction induced by tubulin knockdown. Mock-Tg/rrf-3 and WT4R(L)-Tg/rrf-3 worms were grown on L4440::T, L4440::mec-12 + L4440::T, or L4440::mec-12 + L4440::tau RNAi plates from embryo to 4 days after hatch. **(A)** Expression levels of MEC-12, tau and GAPDH were quantified by Western blotting. Lower panel showed the relative expression of Mec-12 and tau against naive worms (means ± SEM, *n* = 4). **(B)** The average number of responses per 5 trials of anterior touch are shown (means ± SEM, *n* = 80). The touch-sense abnormality of WT4R(L)-Tg/rrf-3 induced by *mec-12* RNAi was significantly reduced by co-administering tau RNAi. Statistical significance was analyzed by Tukey’s *post hoc* test (^∗^*p < 0.05*, ^∗∗^*p < 0.01*, and *^∗∗∗^p < 0.001*).

These data indicate that the relative levels of tau and tubulin are important regulators of tau toxicity. We next tested whether the abnormalities of *mec-12* RNAi worms can be rescued by reducing tau expression. Reduced expression of tau by simultaneous RNAi knockdown significantly rescued touch sense abnormalities induced by *mec-12* RNAi in WT4R(L)-Tg/rrf-3 worms (**Figure 4**). Thus, excessive expression of tau against tubulin is critical for tau-induced neurotoxicity.

**FIGURE 5:**
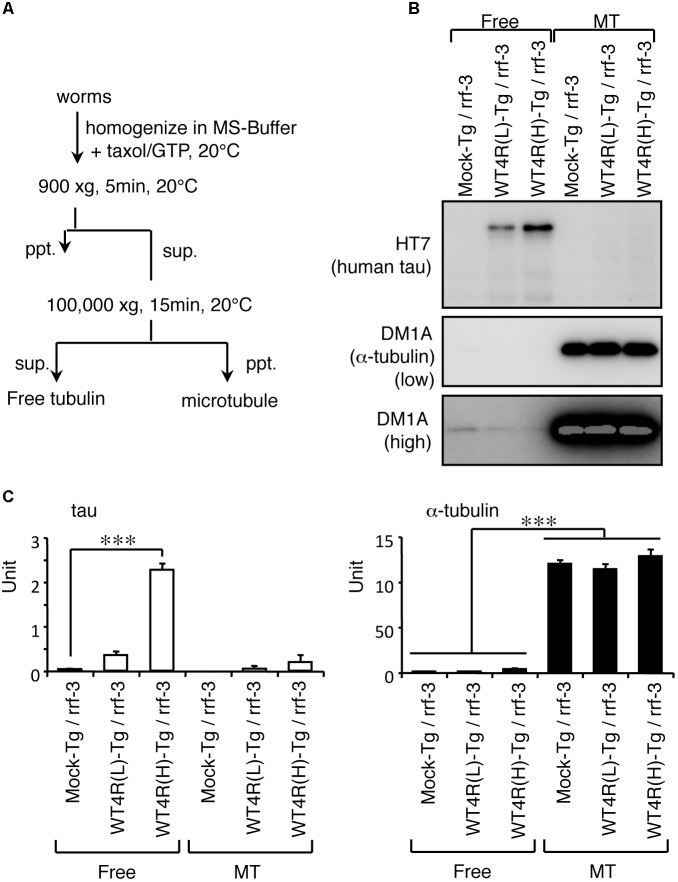
Human-tau in punc-119::tau worms are not bound to MTs. **(A)** Flowchart of fractionation of MT and free tubulin fractions from worms are shown. **(B)** Western blotting of free tubulin (Free) and MT fractions from indicated worms using anti-tau (HT7) and anti-α-tubulin (DM1A) antibodies. **(C)** The amounts of tau and α-tubulin in Free and MT fractions were quantified (means ± SEM, *n* = 5). Statistical significance was analyzed by Tukey’s *post hoc* test (*^∗∗∗^p < 0.001*).

**FIGURE 6:**
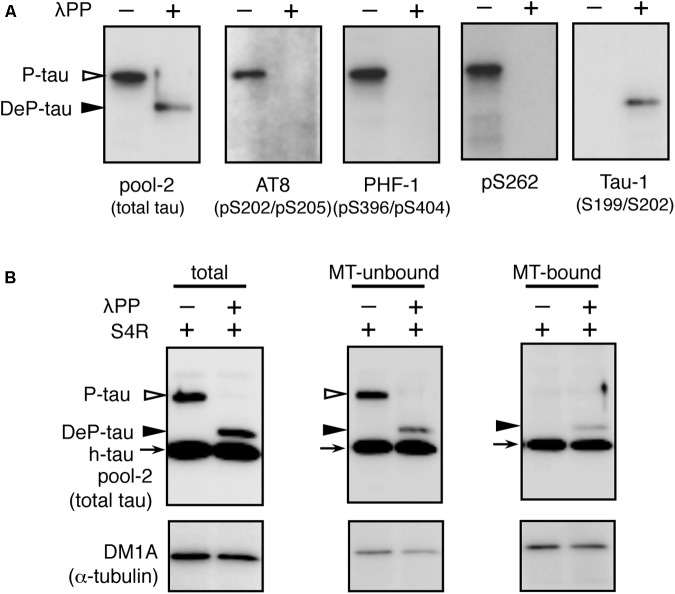
*In vitro* dephosphorylation restores the MT-binding activity of human-tau in tau-Tg worms. **(A)** l-phosphatase dephosphorylation of tau in worms was assessed by Western blotting using the indicated antibodies. **(B)** Purified tau from WT4R(H)-Tg worms were remixed with recombinant human 0N4R tau isoform (h-tau, positive control) and MTs from naive worms (total). MT-unbound and MT-bound fractions were prepared from this mixture as described in the Materials and Methods section. Bands corresponding to phosphorylated tau (P-tau, open arrowheads), dephosphorylated tau (DeP-tau, closed arrowheads), and recombinant tau (h-tau, arrows) are indicated. Note that a portion of dephosphorylated tau, but not phosphorylated tau, was recovered in the MT-bound fraction like recombinant tau.

How does the imbalance of tau and tubulin (or MTs) cause neurotoxicity? We first analyzed the properties of the MT-binding of tau expressed in the transgenic worms. MT-bound tau and free MT-unbound tau were fractionated after stabilizing MTs by Taxol. We found that human-tau, even in WT4R(L)-Tg worms, was almost completely recovered in the MT-unbound fraction (**Figure 5**). Because tau in transgenic worms is phosphorylated to a higher extent similar to PHF-tau (Miyasaka et al., 2016), we suspected that this may be caused by the abnormal phosphorylation. To investigate this, we purified human-tau from tau-Tg worms and tested their MT-binding after dephosphorylation. As shown in **Figure 6**, dephosphorylation by lambda protein phosphatase induced a large electrophoretic mobility shift and resulted in the loss of immunoreactivity for phosphorylation-dependent antibodies. Interestingly, dephosphorylation also resulted in the increased binding of tau to MTs (**Figure 6**). This suggests that the MT itself or MT-binding of tau is not a plausible explanation for how the imbalance of tau and tubulin causes neurotoxicity.

**FIGURE 7:**
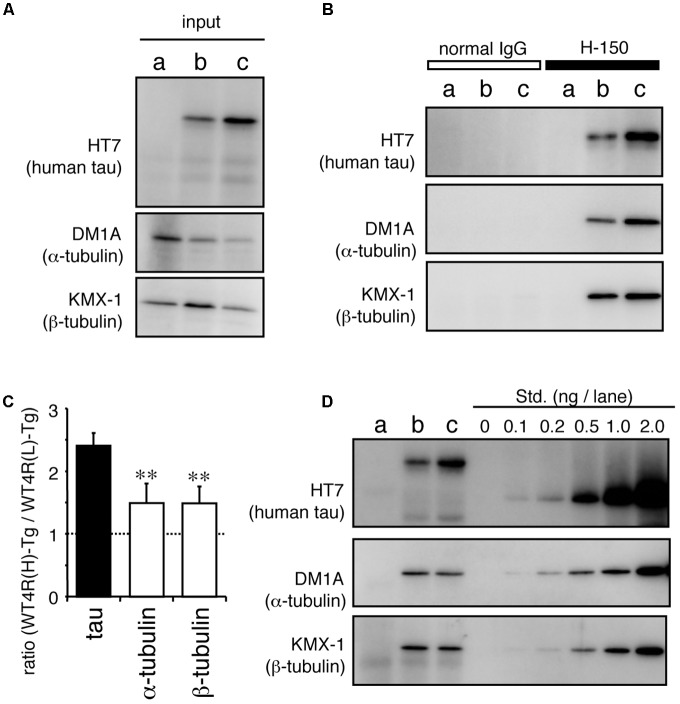
MT-unbound tau in tau-Tg worms interacts with α/β-tubulin dimers in the soluble fraction. MT-unbound soluble fractions were prepared from Mock-Tg **(a)**, WT4R(L)-Tg **(b)**, and WT4R(H)-Tg **(c)** worms and subjected to the immunoprecipitation using anti-tau IgG (H-150). **(A)** Expression of tau, α-tubulin, and α-actin in the total lysate (input) are shown. **(B)** Immunoprecipitated proteins were analyzed using anti-tau (HT7), anti-α-tubulin (DM1A), and anti-β-tubulin (KMX-1) antibodies. **(C)** Relative amount of tau and α/β-tubulin in the co-immunoprecipitaed fraction from tau-Tg worms were quantified (mean ± SEM, *n* = 6). **(D)** Quantitative analysis of each protein indicates that the nearly equal amount of α- and β-tubulin were co-immunoprecipitated with tau. Std. indicates the purified recombinant tau (for HT7) or porcine tubulins (for DM1A and KMX-1). Statistical significance was analyzed by Tukey’s *post hoc* test (^∗∗^*p < 0.01*).

Recently, it has been suggested that tau also interacts with tubulin dimer(s) (Li et al., 2015; Li and Rhoades, 2017). To test whether MT-unbound tau is associated with tubulin dimers, we immunoprecipitated tau from tau-Tg worms and performed Western blotting As shown in **Figure 7**, both α- and β-tubulin were co-immunoprecipitated with soluble tau (**Figure 7**), despite tau being unbound to MTs (**Figure 5**), indicating that tau binds to tubulin dimers. Quantitative analyses showed that the higher amount of tau was recovered from WT4R(H)-Tg than WT4R(L)-Tg. However, the relative amount of co-purified α- and β-tubulins were significantly lower in WT4R(H)-Tg than that of tau. This finding indicatets that free-tau, which does not bind to either MTs or tubulin, may increase when the expression levels of tau are high. Approximately equal proportion of α- and β-tubulins were recovered in tau-bound fraction, suggesting that the α/β-tubulin dimers are associated with soluble tau (**Figure 7D**). The binding between tubulin dimers and MT-unbound tau is also confirmed in the mouse brain (data not shown).

**FIGURE 8:**
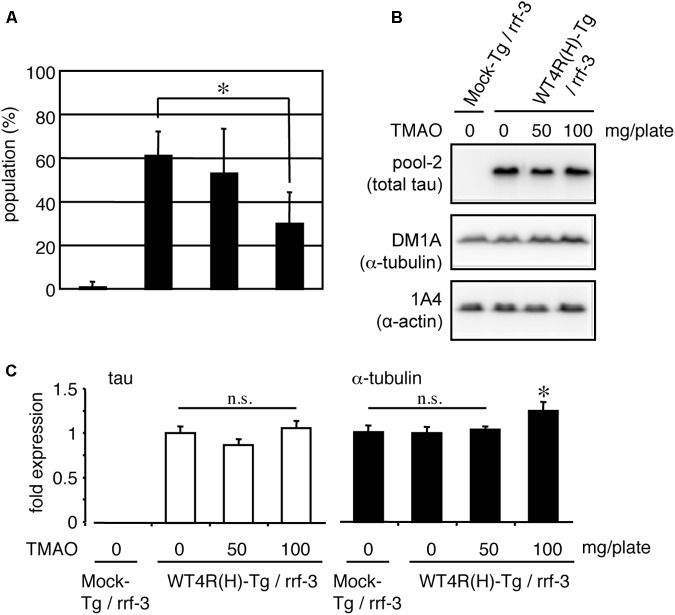
TMAO improves behavioral phenotypes of tau-expressing worms. **(A)** Mock-Tg or WT4R(H)-Tg worms were treated with TMAO for 4 days and evaluated for Unc. The data indicate the populations of severely affected worms (means ± SEM, *n* = 5). **(B)** Total lysates from TMAO treated worms were analyzed by Western blotting using antibodies indicated. **(C)** The amounts of tau and α-tubulin were quantified (means ± SEM, *n* = 7). Statistical significance was analyzed by Tukey’s *post hoc* test (*^∗^p < 0.05*).

The abundance of free-tau in neurons is a possible cause of tau toxicity. If so, it is conceivable that enhanced binding of tau to MT or tubulin may rescue tau neurotoxicity. TMAO is a chemical osmolyte that can stabilize protein conformations and is known to restore the MT-binding ability of phosphorylated tau *in vitro* (Tseng et al., 1999; Smith et al., 2000). WT4R(H)-Tg worms were grown on media containing TMAO and subjected to behavioral and biochemical analyses. As shown in **Figure 8**, the number of Tg worms having the Unc phenotype was significantly reduced by TMAO in a dose-dependent manner (**Figure 8A**). TMAO did not affect the expression of tau. However, high-dose of TMAO slightly enhanced the expression of tubulin (**Figures 8B,C**). Thus, the optimization of protein conformations and/or protein-protein interactions can ameliorate the neuronal dysfunction of WT4R(H)-Tg worms.

**FIGURE 9:**
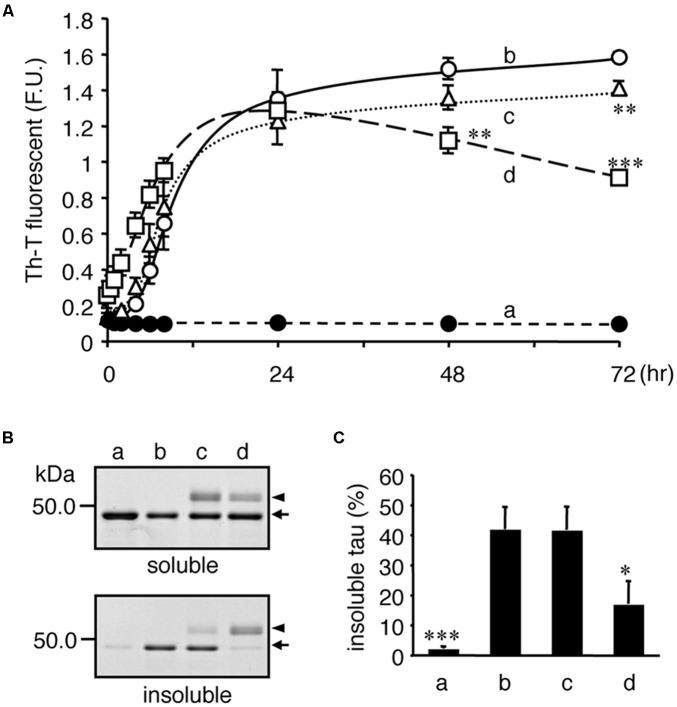
Tubulin inhibits heparin-induced aggregation of tau. Recombinant tau **(a–d)** and tubulin (0.2 mg/ml; **c**, 1.0 mg/ml; **d**) were incubated without (closed: **a**) or with (open: **b–d**) heparin at indicated periods. **(A)** Time courses of Th-T fluorescence change are shown (means ± SEM, *n* = 4). The statistical significance compared to controls (+heparin, 0 mg/mL tubulin) was analyzed by Tukey’s *post hoc* test (^∗^*p < 0.05*, ^∗∗^*p < 0.01*, and *^∗∗∗^p < 0.001*). **(B)** After 72 hincubation, the proteins were subjected to the Sarkosyl-solubility assay as described in the Materials and Methods section. Gel images of CBB staining of total, Sarkosyl-soluble, and Sarkosyl-insoluble fractions are shown. Arrows and arrowheads indicate tau and tubulin, respectively. Note that the Sarkosyl-insoluble tau was reduced in the presence of tubulin (the arrow in lowest panel). **(C)** The amounts of Sarkosyl-insoluble tau were quantified (means ± SEM, *n* = 4). Statistical significance was analyzed by Tukey’s *post hoc* test (^∗^*p* < 0.05, ^∗∗∗^*p < 0.001*).

We further addressed whether tubulin directly attenuates tau aggregation, a typical abnormality identified in tauopathy neurons. As shown in **Figure 9**, recombinant tau formed Th-T-positive insoluble aggregates following co-incubation with heparin (**Figure 9A**). When similar amounts of purified tubulin and tau were added to the mixture, the Th-T values immediately increased. Because MT formations can occur under these conditions, the transient increase in Th-T fluorescence may be caused by MT-formation (Ackmann et al., 2000), but not tau aggregation. However, after 24 h, the Th-T fluorescence decreased and by 48 h, and the fluorescence was significantly lower than control values (**Figure 9A**). After 72 h of incubation, Sarkosyl-insoluble tau, corresponding to the aggregated tau, was verified. **Figure 9B** indicates that the Sarkosyl-insoluble tau emerged only in the presence of heparin. Co-incubation of equivalent tubulin with tau reduced the amount of tau fractionated into the Sarkosyl-insoluble fraction (**Figure 9B**). Although the lower (one-fifth) amount of tubulin did not showed a transient Th-T increase, a weak but significant Th-T reduction was observed at 72 h. Taken together with the TMAO results, these data indicate that enhancing the binding of tau to tubulins may help reduce tau pathology through the reduction of neuronal dysfunction and tau aggregation.

## Discussion

Here, we demonstrated that not only an overexpression of tau but also a reduction of tubulins enhances tau-related pathology in worm models of tauopathy. Neuronal function was consistently rescued by either the suppression of tau expression or the pharmacological stabilization of tau/tubulin-binding. Furthermore, tubulin inhibited tau aggregation promoted by heparin *in vitro*. These data indicate that an imbalanced expression of tau compared to tubulin, specifically an excess of tau, is detrimental.

A loss of MTs and tubulin have been reported in the neurons of AD brains (Terry et al., 1964; Cash et al., 2003; Zhang et al., 2015). Interestingly, MT loss was found in neurons without neurofibrillary tangle formations, suggesting that the MT loss occurs independent of or ahead of the tangle formation. It is well known that tubulins are vulnerable proteins and need energy or co-factors to function properly (Arai et al., 1975). Thus, it is reasonable that the chronic decrease in metabolism, or the increased impairment of the MT protection and/or repair systems, will read the fray of cytoskeleton in the long-lived neurons.

Age is considered as a strong risk factor in neurodegenerative diseases such as AD. Various age-related abnormalities in cellular systems are considered in neurons (Baker and Petersen, 2018), and it is plausible that one or some of them are involved in triggering of tauopathy. Although further analysis is needed, dysregulation of the neuronal cytoskeletal proteins can also be defined as one character of brain protein aging, which may leads to the onset of neurodegenerative diseases. In the natural course of aging, the destruction of MTs has not been identified in nematodes or mice. Considering the lifetime of them, it is reasonable to assume that it takes longer time for this phenomenon to occur. In that sense, we cannot deny the possibility that the age-dependent tubulin/MT loss is a phenomenon unique to the neurons in the human brain that can live for decades.

In physiological condition, a much higher amount of tubulin compared to tau is present in brains. Our preliminary study indicated that there is roughly a 60-fold higher concentration of tubulin than tau in adult mice brains (data not shown). Although, it is difficult to estimate the local concentration of tau and tubulin in each cell type (ex. neuron, glia) or in each subcellular compartment (ex. cell body, axon, and dendrite), the above estimate indicates that slight changes of tau expression or tubulin reduction may not immediately tip their balance. This is consistent with the finding that it takes 8 months to develop pathology even in the PS19 line expressing P301S-tau at a five-fold higher level than endogenous tau (Yoshiyama et al., 2007). Interestingly, in the R406W-tau Tg mice, which have a lower level of tau and show mild pathology only after 18 months of age (Tatebayashi et al., 2002), tau aggregates were sparsely observed in neurons exhibiting tubulin-loss. Therefore, we speculate that tau pathology could result from not only aberrant expression of tau but also tubulin loss in aged neurons. It is also possible that once the aggregation of tau is initiated, MT-degeneration is stimulated (Alonso et al., 1997). Thus, tau aggregation and tubulin degeneration may be developed cooperatively.

Here, we showed an interaction between tau and tubulin dimers in the MT-unbound fraction prepared from worms. This is reproduced in mouse brains (data not shown). Recently, it has been demonstrated that tau can bind to tubulin dimers and form multiple oligomers *in vitro* (Elbaum-Garfinkle et al., 2014; Li et al., 2015; Li and Rhoades, 2017). Because tau binds to the C-terminal region of tubulin at sub-micromolar Kd, it is reasonable that virtually all tau molecules are bound to MT or soluble tubulin dimers in healthy neurons (Fauquant et al., 2011; Lefevre et al., 2011). We could not distinguish by ultracentrifugation if the complex consisted of simple trimers of tau and α/β-tubulin dimers, or multiple complexes like small soluble MTs. However, it is likely that the MT-unbound tau does not behave alone despite its tau/MTs binding ability. Therefore, the regulatory mechanism for tau-MT binding by phosphorylation could be more complicated than has ever been considered (Bodea et al., 2016; Wang and Mandelkow, 2016).

Both of the responsible regions for MT/tubulin-binding and tau-tau binding (for aggregate formation) are located in the C-terminal region of tau containing MT-binding repeats (Gustke et al., 1994; Perez et al., 1996; Goode et al., 1997; Kadavath et al., 2015; Fitzpatrick et al., 2017; Lathuiliere et al., 2017). We also showed that the C-terminal fragment, including the MT-binding repeats, is responsible for the neurotoxicity of tau (Xie et al., 2014). Simply, it is conceivable that the interaction of an unknown factor(s), or tau-itself on a naked MT-binding domain, is crucial for exerting tau toxicity. Thus, as shown here, the binding of tubulin (or MT) on an MT-binding domain of tau, interferes with the undesirable interaction with other factors, including tau-itself. Because the affinity of tau-tau binding is about 20 times higher than that of the tau/tubulin interaction, a sufficient amount of tubulin may be necessary to suppress the neurotoxicity of tau (Lai et al., 2016). If the level of tubulins declines due to aging or metabolic impairment, neurons may become prone to have higher levels of free tau and eventually tau aggregates.

In summary, our data suggested that the imbalanced expression of tau vs. tubulin might be a key step in the pathogenesis of tauopathy, and “free-tau” may be a toxic species of tauopathy. Thus, we propose the following hypothesis. Tau has the potential to be cytotoxic due to its MT-binding repeats. However, in healthy neurons MTs or tubulin dimers bind on the MT-binding repeats, thereby masking the toxicity. However, in some aged neurons in the elderly, a decline of tubulin and/or ectopic expression of tau occur by energy failure, oxidative stress, or amyloid depositions, which need to be experimentally identified. This will result in genuine “free-tau” that has free, exposed MT-binding repeats, which behave as either a toxic factor by itself, or promotes the self-aggregation of tau.

## Author Contributions

TM and YI designed the experiments. TM, YS, and SatY performed the behavioral and biochemical analyses. SawY, EK-N, and SM developed the *C. elegans* models. TM wrote the manuscript.

## Conflict of Interest Statement

The authors declare that the research was conducted in the absence of any commercial or financial relationships that could be construed as a potential conflict of interest.
